# Minimally Invasive Surgery for Colorectal Cancer

**DOI:** 10.31662/jmaj.2020-0089

**Published:** 2021-01-14

**Authors:** Shinichi Yamauchi, Takatoshi Matsuyama, Masanori Tokunaga, Yusuke Kinugasa

**Affiliations:** 1 Department of Gastrointestinal Surgery, Tokyo Medical and Dental University, Tokyo, Japan

**Keywords:** minimally invasive surgery, colorectal cancer, laparoscopic surgery, robotic surgery, transanal minimally invasive surgery

## Abstract

In recent years, minimally invasive surgery for colorectal cancer has seen remarkable improvement. For the laparoscopic surgery for colon cancer, earlier postoperative recovery and reduced hospital stays can be expected compared to those for open surgery. Also, no increase in perioperative morbidity and mortality has been shown. Furthermore, long-term oncological outcomes comparable to open surgery have been obtained. Although laparoscopic surgery for rectal cancer has shown good short-term postoperative outcomes, recent randomized controlled trials could not demonstrate non-inferiority to open surgery with respect to oncological safety. Further studies are required to confirm the impact of robotic surgery on colon and rectal cancer and the appropriate indications for transanal total mesorectal excision for rectal cancer.

## Introduction

Gastrointestinal surgical treatment has been changing with the times, from extended surgery pursuing curability in the past to the standardization of procedures and the focus on function-preserving surgery. In recent years, minimally invasive surgery (MIS) has made remarkable progress. MIS, as in laparoscopic surgery, has been introduced for benign and malignant diseases, and various clinical trials have been conducted on the safety, cosmetic results, and curability achieved through this technique.

Surgical resection is the basis of treatment for colorectal cancer, and the quality of the surgery is an important factor. MIS for colorectal cancer, as with other cancers, has been widespread. Several large randomized controlled trials (RCTs) have shown the equivalence or non-inferiority of MIS to conventional surgery. Thus, open surgery is no longer considered the gold standard. However, some RCTs have not shown equivalence between open surgery and MIS for rectal cancer, suggesting that this surgical technique requires improvement. This situation has led to an increasing focus on novel techniques, including transanal total mesorectal excision (TaTME) and robotic surgery, which may improve the technical problem, and a variety of evidence has been created.

This review article outlines the MIS treatment for colon and rectal cancer, while reviewing the representative clinical studies globally.

## 1. Open vs. MIS for Colon Cancer

### RCT comparing open and laparoscopic colectomy

During the three decades since the first report of laparoscopic surgery for colorectal cancer in 1991, the results of numerous large-scale clinical trials have been reported, and laparoscopic surgery has been establishing its position as a standard operation in the treatment of colorectal cancer ^(1)^.

From the second half of the 1990s to the 2000s, multiple RCTs comparing open and laparoscopic surgeries for colon cancer were conducted (Table 1).

In a 2002 trial of 206 cases from Barcelona, Spain, Lacy et al. indicated that laparoscopic surgery is an independent risk reduction factor for the recurrence rate, cancer death rate, and overall mortality and that this effect could be caused by the poor performance of open surgery for stage III colon cancer. In addition, they also indicated that less invasive laparoscopic surgery may have improved patients’ immune status, slowed tumor progression, and reduced infiltration ^(2), (3)^.

**Table 1.** Randomized Controlled Trials Comparing Open and Laparoscopic Colectomy.

Trial	Ref.	Year of publication	Year of registrasion	Number of cases		Conversion rate	Postoperative complications	5yDFS	5yOS
Barcelona	2, 3	2004	1993-1998	Lap	105	11%	12%	(Overall mortality 36%)	
				Op	101	-	31%*	(Overall mortality 49%)	
COST	4, 5, 6	2007	1995-2001	Lap	435	21%	21%	80%	86%
				Op	428	-	20%	81%	85%
CLASICC	7, 8, 9	2007	1996-2002	Lap	273	25%	26%	58%	56%
				Op	140	-	27%	64%	63%
COLOR	10, 11, 12	2017	1997-2003	Lap	627	17%	21%	67%	74%
				Op	621	-	20%	68%	74%
JCOG0404	13, 14, 15, 16	2017	2004-2009	Lap	529	5.4%	14%	79%	92%
				Op	528	-	22%*	80%	90%

Ref.: number of reference, Lap: laparoscopic colectomy, Op: open colectomy, DFS: desease-free survival, OS: overall survival *: P<0.05

In the Clinical Outcomes of Surgical Therapy (COST) trial, a large-scale RCT conducted in 48 institutions in the United States and Canada analyzing 863 cases accumulated from 1995 to 2001, laparoscopic surgery showed significantly better results in length of hospital stay and analgesic use while showing similar mortality within 30 days after surgery, rates of complications, re-hospitalization, and reoperation. Regarding the long-term prognosis, neither the recurrence rate (21.8% vs. 19.4%) nor the 5-year disease-free survival (DFS) rate showed a significant difference between open and laparoscopic surgery. The rate of conversion to open surgery in the laparoscopic surgery group was as high as 21%, with no difference found in different institutions ^(4), (5), (6)^.

For the Conventional versus Laparoscopic-Assisted Surgery in Colorectal Cancer (CLASICC) trial conducted in 27 institutions in the United Kingdom, 794 colorectal cancer cases (colon and rectal cancer) were registered from 1996 to 2002. Greater safety of the laparoscopic surgery group was suggested, with no significant differences in the postoperative complication rate and mortality between open and laparoscopic surgery for colon cancer. In addition, there was no significant difference in the postoperative 3-year recurrence-free rate and overall survival (OS) rate between the two groups. With a 25% higher rate of conversion to open surgery, however, the DFS rate in cases of conversion was significantly poorer than that of the open and completed laparoscopic surgery groups ^(7), (8), (9)^.

The Colon Cancer Laparoscopic or Open Resection (COLOR) trial was an RCT conducted in 29 institutions in 7 European countries, analyzing 1,076 cases accumulated from 1997 to 2003. The short-term outcomes, such as postoperative pain and hospital stay, were better in the laparoscopic colectomy group than in the open colectomy group. There is no difference in the postoperative complication rate and mortality between the two groups, suggesting the feasibility of laparoscopic surgery for colon cancer. In the long-term outcomes, even though non-inferiority was not proved statistically (because the 3-year DFS rate as a primary endpoint did not exceed the non-inferiority margin in the laparoscopic surgery group), it was concluded that laparoscopic surgery is acceptable for standard care, as the recurrence pattern, DFS, OS, and distant metastasis rate were similar between the two groups ^(10), (11), (12)^.

The Japan Clinical Oncology Group (JCOG) 0404 study was conducted in Japan, and the results were published in 2017. From 2004 to 2009, 1,057 cases of cStage II/III colon cancer (including recto-sigmoid cancer) were registered at 30 facilities in Japan, and the study analyzed the surgical outcomes of the open and laparoscopic approaches. In the short-term results, there were less bleeding, faster recovery of intestinal peristalsis, shorter hospital stays, and fewer surgical site infections in the laparoscopic group compared with the open surgery group ^(13)^. Concerning the long-term results, the 5-year OS rate was 90.8% for the open and 91.8% for the laparoscopic group. However, the non-inferiority of laparoscopic surgery was not verified. The improved outcomes were considered because the treatment results were better than expected in both groups and the events number reaching only half of the planned number. In any case, laparoscopic surgery became an acceptable surgical option ^(14), (15), (16)^.

According to one meta-analysis of multiple RCTs (including the above trials), the non-inferiority of laparoscopic surgery to open surgery in OS has been demonstrated (hazards ratio [HR] = 0.94, 95% confidence interval [CI]: 0.80-1.09) ^(17)^. Moreover, it is verified that there was no difference in technical and oncological safety. However, considering the large-scale RCT data, the laparoscopic group had a high conversion rate, which appeared to be associated with increased postoperative complications, mortality, blood transfusions, and recurrence, representing an important clinical problem ^(18), (19)^. The safety and efficacy of laparoscopic surgery with conversion for high-risk groups, such as obese patients, and for high-difficulty transverse colon cancer that has been excluded from RCTs have not been fully established. There is scope for further investigation and consideration in the future.

### Robotic surgery for colon cancer

Robotic surgery is characterized by high-resolution three-dimensional images, forceps with a wide motion range, and functions including image stabilization and motion scaling. In addition, this system enables highly flexible, accurate, and precise surgery and is expected to overcome problems in laparoscopic surgeries, thereby becoming popular globally. Since the first report on robotic surgery for colorectal cancer in 2002, several studies have reported its safety and feasibility ^(20), (21), (22), (23)^. However, almost all of these reports are from retrospective studies; the only report from an RCT was from a 2009 study with a limited number of cases, and it found no difference in short- or long-term outcomes between robotic and laparoscopic colectomy ^(24)^. Recently, discussions based on a large database instead of on RCTs have been reported. Specifically, Kulaylat et al. and Schootman et al. have compared the data of the American College of Surgeons (ACS) National Surgical Quality Improvement Program (2013-2015) on robotic (3,864 cases and 2,233 cases, respectively) and laparoscopic colectomy (40,063 cases and 10,844 cases, respectively) after adjusting the selection bias according to propensity scores. They reported a lower rate of conversion (6.0% vs. 11.5%, P < 0.00133; 5.7% vs. 18.8%, P < 0.0534) and a shorter hospitalization period (4.6 days vs. 5.2 days, P < 0.001) for robotic colectomy ^(25), (26)^.

Robotic colectomy is not covered by the health insurance scheme in Japan, resulting in fewer operations and less evidence for colectomy than for resection of rectal cancer. Currently, based on information from those countries utilizing robotic surgery, a prospective study of robotic surgery for colon cancer (Japan Registry of Clinical Trials [jRCT] 1032190036: “evaluation for the safety of robotic-assisted colectomy for rectal colon cancer: a multi-institutional, prospective, historical controlled, feasibility study”) is being conducted. Researchers in this study expected that Japan will generate evidence for the method’s safety, feasibility, and ability to achieve a cure.

## 2. Open vs. MIS for Rectal Cancer (Table 2)

The CLASICC trial is the first RCT to compare open and laparoscopic surgery for rectal cancer. Laparoscopic surgery showed superior results regarding the postoperative hospital stay compared with open surgery, while the postoperative complication rate was similar in both the laparoscopic and open surgery groups. The primary endpoint of the circumferential resected margin (CRM)-positive rate was higher in the laparoscopic surgery group at 12% than in the open surgery group at 6%, although the difference between the groups was statistically insignificant. No difference was observed in the 3- and 5-year local recurrence, recurrence-free survival, and OS ^(7), (8), (9)^.

**Table 2.** Randomized Controlled Trials Comparing Open, Laparoscopic, and Robotic Resection of Rectal Cancer.

Trial	Ref.	Year of publication	Year of registrasion	Number of cases		Conversion rate	Postoperative complications	Positive CRM	Local recurrence rate	DFS	OS
CLASICC	7, 8, 9	2007	1996-2002	Lap	235	34%	40%	12%	11%	53%	60%
				Op	128	-	37%	6%	9%	52%	53%
									(5y)		
COLOR II	27, 28	2015	2004-2010	Lap	699	17%	40%	10%	5%	75%	87%
				Op	345	-	37%	10%	5%	71%	84%
									(3y)		
COREAN	29, 30	2014	2006-2009	Lap	170	1.2%	21%	2.9%	2.6%	79%	92%
				Op	170	-	24%	4.1%	4.9%	73%	90%
									(3y)		
ACOSOG Z6051	31, 32	2019	2008-2013	Lap	240	11%	23%	12%	4.6%	80%	-
				Op	222	-	22%	7.7%	4.5%	83%	-
									(2y)		
ALaCaRT	33, 34	2019	2010-2014	Lap	238	9%	19%	7%	5.4%	80%	-
				Op	237	-	26%	3%	3.1%	82%	-
									(2y)		
ROLARR	38, 39	2017	2011-2014	Lap	234	12.2%	32%	6.3%	-	-	-
				Robo	237	8.1%	33%	5.1%	-	-	-

Ref.: number of reference, Lap: laparoscopic rectal resection, Op: open rectal resection, Robo: robotic rectal resection, DFS: desease-free survival, OS: overall survival

### RCT comparing open and laparoscopic rectal resection

In the COLOR II trial, an RCT conducted in 30 institutions in eight European countries by analyzing 1,044 cases accumulated from 2004 to 2010, no significant differences in intra- and postoperative complications and mortality were observed between the open and laparoscopic surgery groups, and the primary endpoint of the 3-year local recurrence rate of 5% in both groups proved the non-inferiority of the laparoscopic group to the open surgery group. The 3-year DFS and OS rates were also similar in the two groups ^(27), (28)^.

The Comparison of Open versus Laparoscopic Surgery for Mid and Low Rectal Cancer After Neoadjuvant Chemoradiotherapy (COREAN) trial, an RCT in Korea that analyzed 340 cases registered from 2006 to 2009, examined open and laparoscopic surgeries for rectal cancer after preoperative chemoradiotherapy. The primary endpoint of the 3-year DFS rate was 72.5% (95% CI: 65.0-78.6) in the open and 79.2% (72.3-84.6) in the laparoscopic surgery group, showing a difference of 6.7% between the groups. As the lower limit of the confidence interval (95% CI: −15.8-2.4) was inferior to the non-inferiority margin (p < 0.0001), the non-inferiority was proved. No difference was observed between the groups in OS rate, local recurrence rate, and quality of life, among these secondary outcomes ^(29), (30)^.

The ACS Oncology Group (ACOSOG) Z6051 trial, conducted in 35 institutions in the United States and Canada, compared open and laparoscopic surgeries for stage II/III rectal cancer. The success rate of the primary endpoint of completeness of TME (success in pathological surgery) was 81.7% and 86.9% in the laparoscopic and open surgery groups, respectively, and resulted from reviews of the 262 cases accumulated from 2008 to 2013. As the difference between the groups was −5.3%, and the lower limit of its 95% CI was −10.8% (P = 0.41), its non-inferiority was not proved ^(31), (32)^.

The Australian Laparoscopic Cancer of the Rectum Randomized Clinical Trial (ALaCaRT), conducted in 24 institutions in Australia and New Zealand, analyzed the 473 cases registered from 2010 to 2014 and compared the appropriate surgical resection rate between the open and laparoscopic surgery groups. Although appropriate surgical resection was achieved in the open and laparoscopic surgery groups at respective rates of 89% and 82%, statistical non-inferiority was not proved for the laparoscopic surgery group. In addition, a higher CRM-positive rate of 7% was observed in the laparoscopic surgery group, compared with 3% in the open surgery group ^(33), (34)^.

Of the above four RCTs, the latter two trials indicated that CRM positivity tended to be inferior in the laparoscopic group, compared to that in the open group. Laparoscopic surgery for rectal cancer is considered more difficult than that for colon cancers, and the results of two trials indicate that laparoscopic surgeries for rectal cancers require more specialized skills. By contrast, Acuna et al. recently conducted a meta-analysis of 14 RCTs to test the non-inferiority of laparoscopic to open surgery for quality of surgical resection, with a non-inferiority margin based on the consensus of 58 experts worldwide ^(35)^. Consequently, although no conclusions were reached regarding the composite endpoint of the resection success rate between the open and laparoscopic groups, non-inferiority was demonstrated in the rates of positive surgical resection margins, incomplete TME, and positive distal resection margins. The oncological safety and superiority of laparoscopic over open surgery is still under evaluation. However, pathological factors, such as the rate of positive margins, have yielded comparable results; these factors have been shown to influence the long-term prognosis.

### RCT comparing laparoscopic and robotic rectal resection

Robotic surgery can provide the delicate operation for the rectum, which is located in a narrow pelvic cavity and surrounded by functionally important nerves and blood vessels, and can provide excellent preservation of function, such as a reduction in postoperative urinary dysfunction after resection of rectal cancer ^(36), (37)^.

Meanwhile, the result of an RCT of robotic and laparoscopic surgeries for rectal cancer was provided by Jayne et al. in 2017. The study enrolled 1,276 patients operated by 40 surgeons from 29 centers in 10 countries worldwide between 2011 and 2014, of whom 471 patients were randomly assigned to the laparoscopic and robotic surgery groups to investigate the superiority of robotic over laparoscopic surgery. There were no statistically significant differences between the two groups in the primary endpoint of the conversion rate, which was 12.2% in the laparoscopic group and 8.1% in the robotic group. The study concludes that there was no significant difference between the two groups in the primary endpoint ^(38), (39)^.

The subgroup analysis revealed that the rate of conversion of the robotic group was significantly lower in males, obese patients, and relatively difficult surgeries such as low anterior resection, indicating the value of robotic surgery for difficult cases. Additionally, the sensitivity analysis of the effect of the learning curve for robotic surgery indicated that a larger number robotic surgeries performed by the doctor reduced the rate of conversion more than the number of laparoscopic cases did. The results of the study suggest that the clinical benefits of robotic surgery may be limited. However, if the disadvantages are overcome by future innovations and reduced costs, robotic rectal cancer surgery could be further developed.

### Other evidence for MIS for rectal cancer

Evidence from Japan is gradually accumulating, despite the lack of results of large-scale comparative studies. Yamaguchi et al. reported the superiority of robotic surgery for rectal cancer in a high-volume single-center retrospective study of 551 patients with lower- and mid-rectal cancers. In the short-term outcomes of this study, a 0% conversion rate and 1.1% rate of positive surgical resection margins were seen. In the 204 patients in whom long-term outcomes could be provided, the local recurrence rate was only 0.5% ^(40)^.

While the equivalence and superiority of laparoscopic and robotic surgeries to open surgery remain controversial, TaTME is a remarkable surgical technique. In 2016, Penna et al. reported the results of 720 cases treated by TaTME, with a positive CRM rate of 2.4%. Thus, an increase in the rate of radical resection with TaTME is expected ^(41)^. Recently, RCTs comparing laparoscopic TME and TaTME, such as the COLOR III trial and the French Research Group of Rectal Cancer Surgery (GRECCAR) 11 trial, have been conducted, and results of these trials are awaited ^(42), (43)^.

## Conclusion

In this review, we focused on RCTs and outlined the consensus on MIS compared to open surgery for colorectal cancer. Laparoscopic colectomy is established as a standard approach and is associated with better short-term outcomes and equivalent pathological and long-term oncological outcomes to open colectomy. The non-inferiority of laparoscopic to open surgery in terms of pathologic outcomes, local RFS, and other long-term outcomes remain to be proven. Further studies are required to confirm the impact of robotic surgery for colon and rectal cancer and the appropriate indications of TaTME for rectal cancer.

## Article Information

### Conflicts of Interest

None

### Acknowledgement

We would like to thank Editage (www.editage.com) for English language editing.

### Contributions of Authors

SY, TM, MT, and YK contributed to concept, manuscript writing and editing.
